# The impact of a quality management program for patients undergoing head and neck resection with free-flap reconstruction: longitudinal study examining sustainability

**DOI:** 10.1186/s40463-020-00437-2

**Published:** 2020-06-23

**Authors:** Joseph C. Dort, Khara M. Sauro, Shamir Chandarana, Christiaan Schrag, Jennifer Matthews, Steven Nakoneshny, Vida Manoloto, Tanya Miller, C. David McKenzie, Robert D. Hart, T. Wayne Matthews

**Affiliations:** 1 Section of Otolaryngology Head & Neck Surgery, Department of Surgery, University of Calgary Cumming School of Medicine, 3280 Hospital Drive NW, Calgary, Alberta T2N 4Z6 Canada; 2 Ohlson Research Initiative, Arnie Charbonneau Research Institute, University of Calgary Cumming School of Medicine, Calgary, Alberta Canada; 3 Foothills Medical Centre, Alberta Health Services, Calgary, Alberta Canada; 4 Department of Community Health Sciences, University of Calgary Cumming School of Medicine, Calgary, Alberta Canada; 5 Section of Plastic and Reconstructive Surgery, Department of Surgery, University of Calgary Cumming School of Medicine, Calgary, Alberta Canada

**Keywords:** Head and neck cancer, Care pathways, Clinical pathways, Head and neck surgery, Clinical outcomes improvement, Quality improvement, Healthcare delivery

## Abstract

**Background:**

Care pathways (CPs) are helpful in reducing unwanted variation in clinical care. Most studies of CPs show they improve clinical outcomes but there is little known about the long-term impact of CPs as part of a sustained quality management program. Head and neck (HN) surgery with free flap reconstruction is complex, time-consuming and expensive. Complications are common and therefore CPs applied to this patient population are the focus of this paper. In this paper we report outcomes from a 9 year experience designing and using CPs in the management of patients undergoing major head and neck resection with free flap reconstruction.

**Methods:**

The Calgary quality management program and CP design is described the accompanying article. Data from CP managed patients undergoing major HN surgery were prospectively collected and compared to a baseline cohort of patients managed with standard care. Data were retrospectively analyzed and intergroup comparisons were made.

**Results:**

Mobilization, decannulation time and hospital length of stay were significantly improved in pathway-managed patients (*p* = 0.001). Trend analysis showed sustained improvement in key performance indicators including complications. Return to the OR, primarily to assess a compromised flap, is increasing.

**Conclusions:**

Care pathways when deployed as part of an ongoing quality management program are associated with improved clinical outcomes in this complex group of patients.

## Introduction and background

In the article that accompanies this paper we outline the importance of head and neck cancer and the critical role that surgery plays in its management. Surgery is often lengthy [1] and fraught with complications [2–5]. Complexity and complications frequently result in prolonged length of hospital stay (LOS) and are a major driver of hospital costs [6].

Seminal work starting in the 1990s showed the impact of clinical care pathways in improving the quality and cost of care in a broad range of conditions including head and neck cancer surgery. Applying well-known principles of quality management can therefore be an effective strategy to reduce inappropriate variation in care delivery and improve the quality and cost of care [5–13]. Maintaining high clinical performance over time requires ongoing focus and commitment [14]. However, previous studies of care pathways in head and neck surgery report short term outcomes and do not show the impact of a sustained quality management program on long-term outcomes. Therefore, the objective of this study is to evaluate the impact of integrating an evidence-based, expert-informed clinical care pathway on patient outcomes and to evaluate adherence to the pathway. We report patient outcomes that were prospectively collected over a 7 year period and compare these to outcomes collected in baseline (pre-pathway) and early pathway groups.

## Materials and methods

### Intervention & context

The methods and processes to develop and integrate the head and neck surgery clinical outcomes assessment program (the Calgary Program) at the Foothills Medical Centre, Calgary, Alberta, Canada from 2010 to 2012 are described elsewhere.

The Calgary Program encompasses head and neck surgical patients referred to a tertiary care centre in the largest city in the province of Alberta, Canada. Alberta has a single payer and publicly funded healthcare system. The referral population for the Calgary Program is approximately 2 million and over 300 head and neck surgeries are performed each year. Major resection with free flap volumes vary between 50 and 60 cases per year during the time of this study. The program is staffed by 4 Otolaryngologist – Head & Neck surgeons, 3 Plastic and Reconstructive surgeons and is supported by residents and fellows in Otolaryngology and Plastic Surgery. Patients undergoing major resection with free flap reconstruction are managed overnight in the intensive care unit (ICU) and then transferred to a ward staffed by clinicians specialized in managing head and neck surgical patients.

The Calgary Program pathway guides the care of patients undergoing head and neck resection with free flap reconstruction. The pathway is fully integrated into the inpatient electronic medical record (EMR) and as such provides clinical decision support to care providers for these patients. A standard set of orders is initiated by the care provider after a major head and neck surgical procedure. A visual description of the pathway is also provided to the patient/family before, and after, admission to the hospital. While the pathway is standardized, it is also flexible and responsive to individual patient needs and providers can unselect order set elements that are not relevant for a particular patient.

### Cohort

**Fig. 1:**
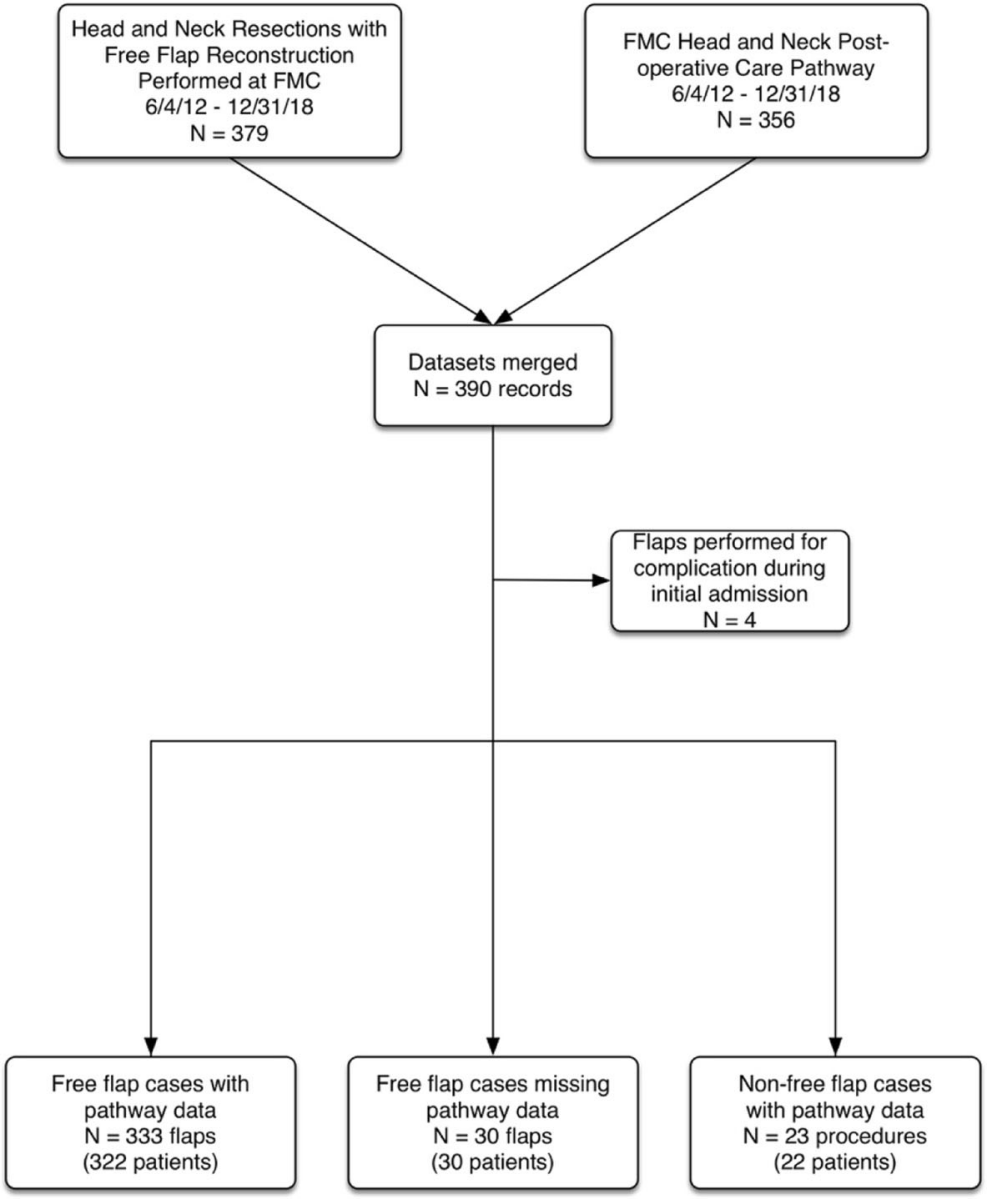
Diagram illustrating cohort composition

All patients undergoing head and neck resection with free flap reconstruction from Jun 4, 2012 until December 31, 2018 were included in this study. The details of this cohort are shown in Fig. 1. Patients treated for head and neck malignancies, benign tumours or complications of other treatments (e.g., osteoradionecrosis) were included. Data from three patient groups are reported: 1) current pathway group (*n* = 333), 2) early pathway group (*n* = 53) and 3) baseline (pre-pathway) group (*n* = 48). The current pathway group comprise patients who were managed from June 2012 until December 2018. These patients were managed under the fully implemented pathway with a mature measurement, audit and feedback system and computerized order sets. The early pathway group includes those managed from 2010 – May 2012 using an earlier version of the pathway. The pre-pathway baseline group includes those whose data were collected from 2005 to 2009 before development and integration of the care pathway.

### Data collection and analysis

Clinical data were collected prospectively by direct care providers and entered into the EMR. Physician clinical notes are currently entered into a paper chart. Clinical data not captured in the EMR were prospectively collected by trained research staff. Retrospective chart review was also conducted to collect additional clinical data on sentinel events, including: return to the operating room or the intensive care unit and prescription of a new medication that is not part of the standard clinical pathway.

Adherence to the pathway components, also called compliance, was measured using the eight key performance indicators (KPI): time to arrival on unit, mobilization time, time to start tube feeds, removal of catheters and tracheotomy tube, and length of stay. Data to measure KPIs were obtained through retrospective chart review, which included physician, nursing and allied health professional notes and diagnostic imaging reports. Adherence to the pathway over time (quarterly from 2012 to 2018) was evaluated by plotting the mean proportion of patients who received pathway concordant care over time. Because of differences in data collection during the different time periods, only mobilization, time to decannulation and LOS can be directly compared among the baseline, early pathway and current pathway groups.

Data were analyzed (Stata v15, Stata Corp, College Station, TX, USA) using proprietary algorithms developed to monitor and evaluate patient care in the Calgary Program. Current pathway data are reported using descriptors such as rates and proportions. Where appropriate intergroup comparisons are made among baseline, early pathway and current pathway groups. These comparisons were made using either Chi squared or Fisher’s exact tests for categorical variables. Continuous variables were compared using Student’s t-test or the Mann-Whitney U test as appropriate. Intergroup comparisons were conducted using one-way ANOVA. In all cases a *p*-value of less than 0.05 indicated a statistically significant result.

## Results

### Cohort

Figure 1 is a diagram that defines the current pathway analysis cohort. We report data from 322 patients who underwent 333 flap procedures. No patients were excluded but in 30 cases pathway data were inadvertently not collected and in 22 cases pathway data were collected on patients who did not meet inclusion criteria. Missing pathway data occurred in less than 10% of the cases and was due to random failure to appropriately flag a case for data collection.

### Patient characteristics

The clinical characteristics of the 3 groups are shown in Table 1. There were no significant differences between the 3 groups with the exception of histology – there was a higher proportion of patients with non-squamous cell cancers in the current pathway group (Table 1).

**Table 1:** Patient Characteristics

Characteristic	Number of cases (%)			^*^p-value
	Baseline (2005–2009) ***n*** = 48	Early pathway (2011–2012) ***n*** = 53	Current pathway (2012–2018) ***n*** = 333	
**Gender**				
Male	32 (67%)	35 (66%)	233 (70%)	ns
Female	16 (33%)	18 (34%)	100 (30%)	
**Age (yrs)**				
Mean (SD)	63.5 (13.2)	62.2 (12.1)	61.7 (12.2)	ns
Range	22.9–84.9	32.8–88.0	21.2–89.0	
**Alcohol consumption**				ns
Never Drinker	11 (23%)	8 (15%)	68 (20%)	
Ex Drinker	4 (8%)	4 (8%)	81 (24%)	
Current Drinker	25 (52%)	36 (68%)	40 (12%)	
Not Reported	8 (17%)	5 (9%)	51 (15%)	
**Smoking status**				ns
Never Smoked	17 (35%)	15 (28%)	83 (25%)	
Ex Smoker	15 (31%)	17 (32%)	108 (32%)	
Current Smoker	12 (25%)	18 (34%)	100 (30%)	
Not Reported	4 (8%)	3 (6%)	42 (13%)	
**Comorbidities**				ns
Diabetes	3 (6%)	5 (9%)	43 (13%)	
COPD	9 (19%)	12 (23%)	41 (12%)	
Hypertension	23 (48%)	23 (43%)	139 (42%)	
Heart Disease	11 (23%)	–	52 (16%)	
**Primary site**				ns
Oral Cavity	40 (83%)	42 (79%)	222 (67%)	
Skin	1 (2%)	1 (2%)	31 (9%)	
Paranasal Sinus	2 (4%)	2 (4%)	22 (7%)	
Other Site	5 (10%)	8 (15%)	58 (17%)	
**Histology**				0.003*
Squamous Cell	44 (92%)	48 (91%)	264 (80%)	
Other Cancer	4 (8%)	2 (4%)	52 (16%)	
Benign	0 (0%)	1 (2%)	17 (5%)	
Not Reported	0 (0%)	2 (4%)	0 (0%)	
**Clinical stage**				ns
0	1 (2%)	3 (6%)	8 (2%)	
I	1 (2%)	6 (11%)	29 (9%)	
II	5 (10%)	6 (11%)	47 (14%)	
III	8 (17%)	9 (17%)	36 (11%)	
IV	25 (52%)	24 (45%)	163 (49%)	
Not Stated	8 (17%)	5 (9%)	50 (15%)	
**Number of free flaps**				ns
1	46 (96%)	53 (100%)	313 (94%)	
2	2 (4%)	0 (0%)	20 (6%)	

^*^Chi-squared or Fisher’s exact test as appropriate

### Key performance indicators

**Fig. 2:**
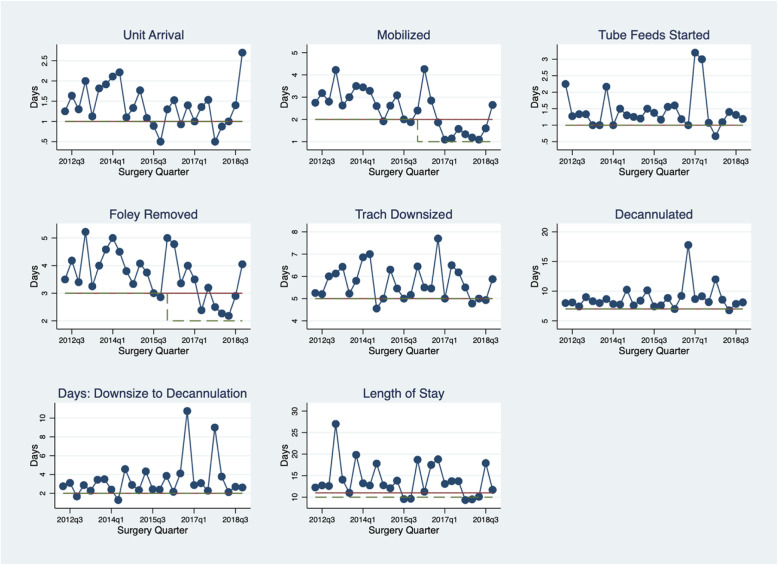
Dashboard of Quarterly Key Performance Indicators

Figure 2 shows a dashboard of 8 KPIs that are routinely reported to the clinical team. The data shown in this figure are quarterly outcomes (means) from Q3 2012 to Q4 2018. KPIs can also be displayed at higher or lower resolution as required by the clinical team. The horizontal solid line represents the median for the entire period and the horizontal dashed line is the target for the displayed KPI. In most cases the median and the target lines overlap, indicating that median performance and the KPI target are congruent. KPIs are variable from quarter to quarter and this is to be expected since some patients are more likely to meet the KPI target than others.

**Fig. 3:**
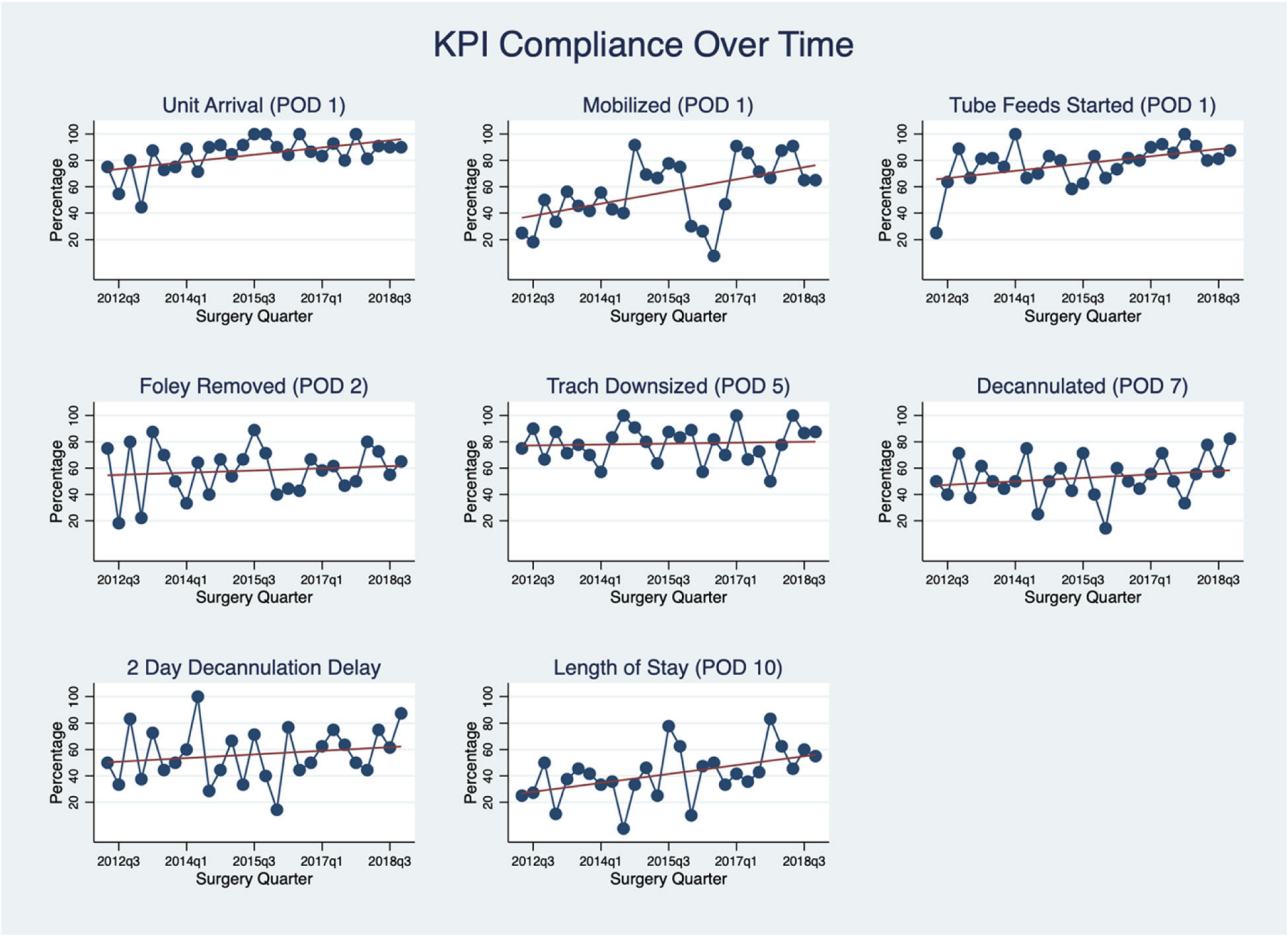
KPI Compliance (proportion of patients achieving KPI goal)

Figure 3 illustrates pathway compliance – the proportion of patients achieving a KPI over time. The solid line is a trend line showing change in the KPI over the duration of the study. In all cases overall performance is either stable or improving over time.

As noted above, 3 KPIs (mobilization time, decannulation time and length of stay) could be directly compared between the 3 groups. These outcome comparisons are shown in Table 2. Both early and current pathway outcomes were significantly improved compared to baseline while no differences were found between the 2 pathway groups.

**Table 2:** Intergroup KPI comparisons

Characteristic	Baseline (***n*** = 61)	Early pathway (***n*** = 55)	Current pathway (n = 333)	^*^p-value
Time to Mobilization (mean days (sd))	4.7 (3.8)	2.5 (0.85)	2.4 (2.8)	0.001
Time to Decannulation (mean days (sd))	13.8 (9.4)	8.2 (3.1)	8.6 (5.2)	0.001
Length of Stay (mean days (sd))	21.6 (17.1)	14.2 (7.1)	14 (11.6)	0.001

^*^ANOVA comparison to baseline. There were no differences between early and current pathway

**Fig. 4:**
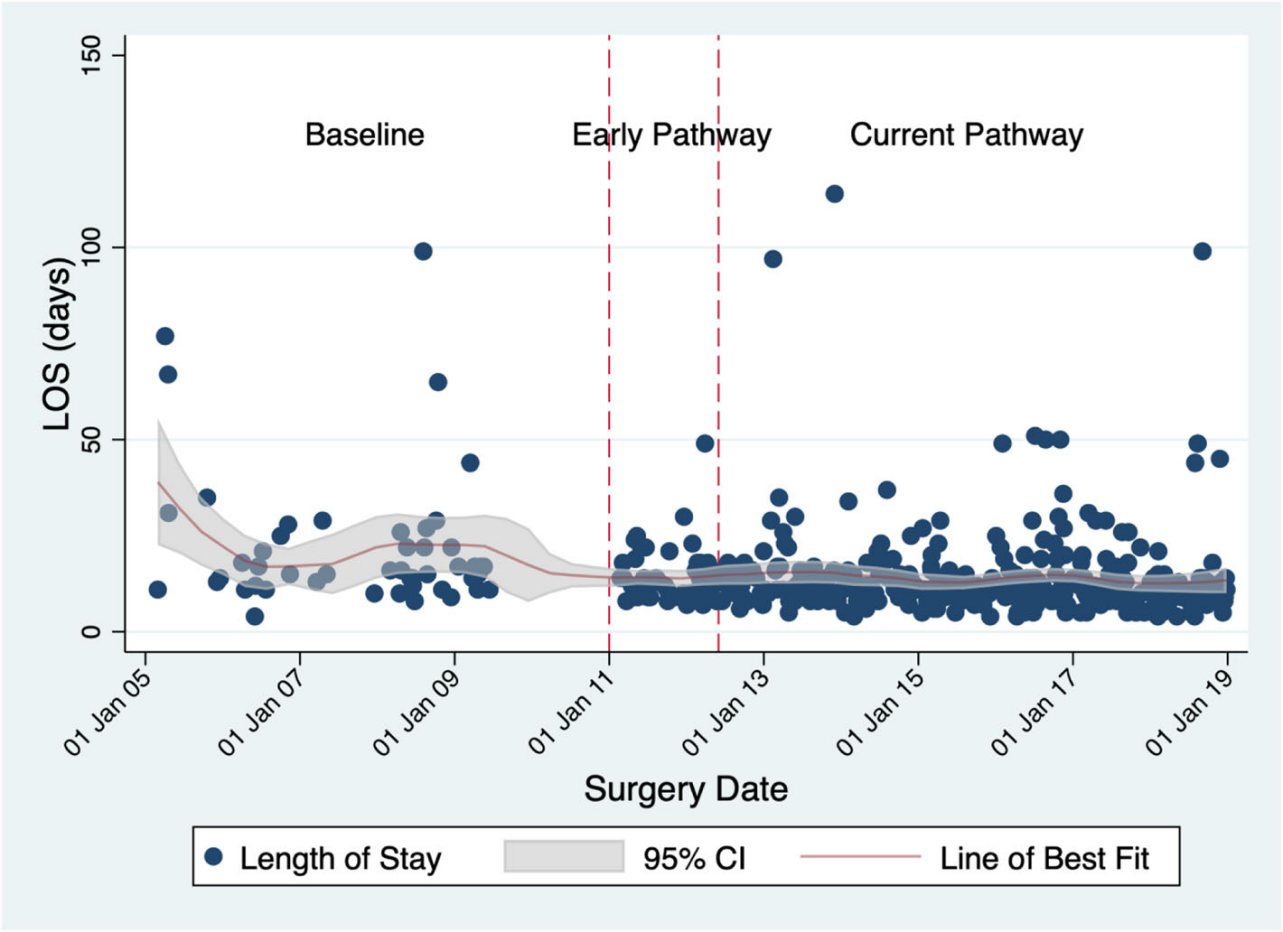
Change in Length of Stay over Time

Detailed length of stay data are shown in Fig. 4, which displays raw data for the baseline, early pathway and current pathway groups. The line of best fit with confidence interval illustrates the downward trend in LOS and also narrowed confidence intervals indicating less variation in LOS in the current pathway group. As shown in Table 2, mean LOS in both pathway groups is consistently shorter than baseline LOS by 7.6 days (*p* < 0.001).

### Complications

**Fig. 5:**
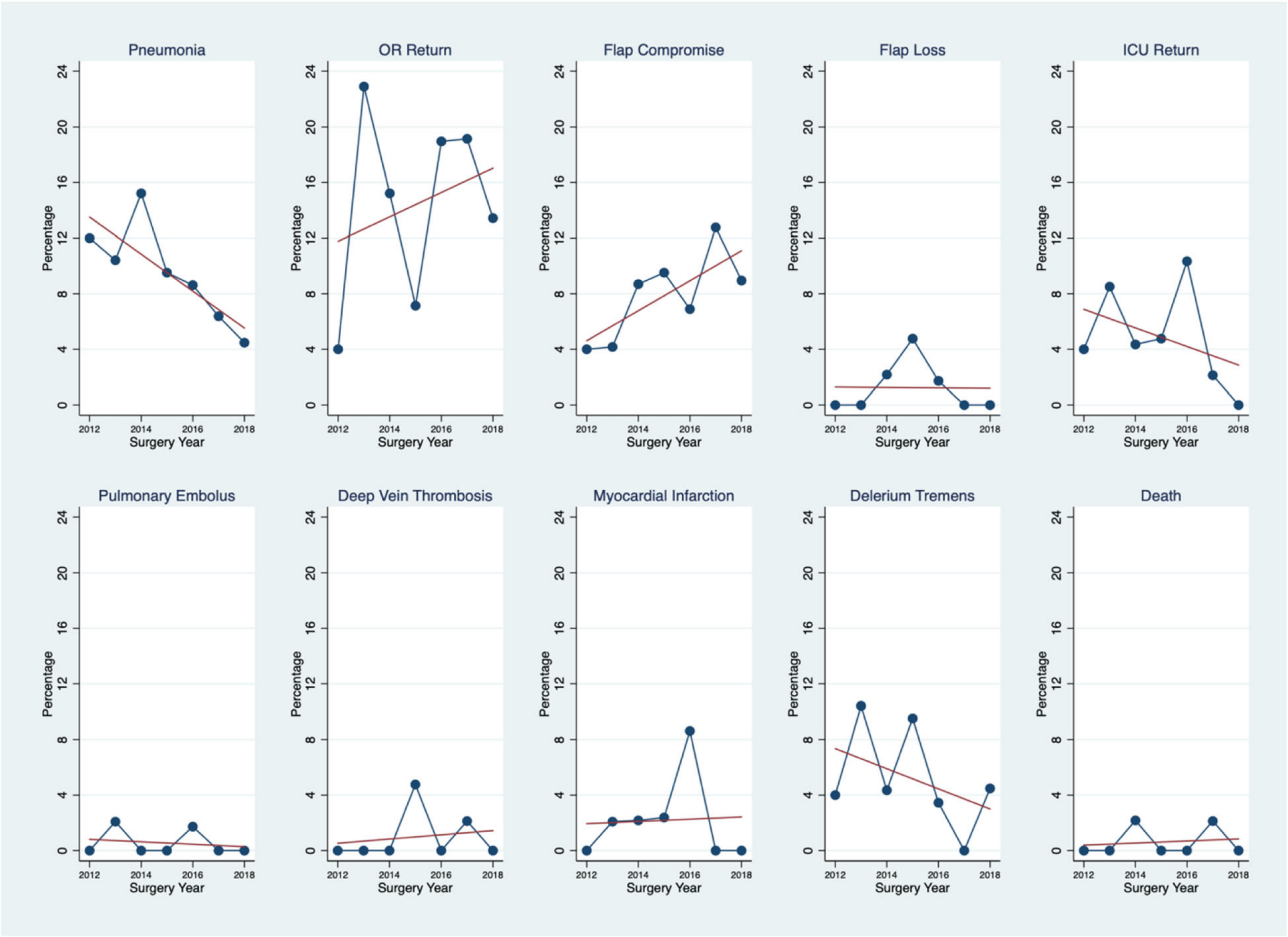
Change in Complications over Time

Figure 5 shows outcomes and annual trends for 10 important complications that are routinely tracked. The largest changes over time are found in pneumonia, readmission to ICU and delirium tremens outcomes. These complications have significantly decreased since the quality management program was implemented. Return to the OR and flap compromise, on the other hand, have increased over the duration of the program. However, flap failure has remained very uncommon. Perioperative death is rare, occurring in 2 patients over the entire study period.

## Discussion

In our companion paper we outlined a practical approach to designing and implementing a quality management program for patients undergoing major head and neck surgery with free flap reconstruction. In this study we report 7 years of prospective outcomes data that demonstrate sustained high clinical performance when compared to a non-pathway managed cohort. Hospital length of stay (LOS), an important measure of overall clinical performance, is consistently lower than baseline and there is no evidence that shorter LOS has an adverse impact on readmission rates or emergency department visits [9]. Due to differences in data collection it was not possible to compare all KPIs between the 3 groups. Data on time to mobilization, decannulation time and length of stay (Table 2), when compared to baseline, were consistently improved. On the other hand complications such as return to the OR and flap compromise appear to be worsening over the duration of the program (Fig. 5). After careful review this apparent decline in clinical performance was primarily due to the use of implantable doppler devices to monitor free flaps. The implantable doppler technology has a higher “false alarm” rate that often makes it necessary to return patients to the OR to check on the vascular anastomosis integrity. This interpretation is supported by the fact that the free flap loss rate has remained stable over the duration of the program with no lost flaps for the last 2 years of the reporting period.

Other authors have demonstrated successful improvement in clinical outcomes through the use of care pathways. Early work by Cohen and colleagues reported significant improvements in clinical, financial and team performance in a diverse cohort of head and neck surgery patients [8]. Later work by Husbands et al. corroborated Cohen’s findings but this paper did not provide much information on the actual pathway that was used [11]. Financial performance is not consistently impacted by the use of care pathways. Chen et al. showed a significant reduction in hospital length of stay in a diverse group of patients undergoing neck dissection but not free flap reconstruction [7]. Furthermore in this study only 30 pathway patients were enrolled and were selected based on surgeon preference.

The new millennium ushered in an era of increased interest in care pathways for head and neck oncologic procedures [7, 10, 13, 15–18]. These studies reported results from a variety of head and neck surgical procedures and some included patients undergoing major resection with free flap reconstruction. Reported benefits included reduced complications, better team dynamics and reduced costs of care. One study by Yueh et al. compared outcomes from 2 different hospitals and concluded that pathways did not seem to have a beneficial impact on LOS [18]. This study however excluded complicated patients therefore it is difficult to interpret the findings. Studies from the Calgary group showed that care pathways were associated with improved clinical and financial performance as well as reductions in post-discharge healthcare utilization [5, 6, 9, 19]. A systematic review of head and neck care pathways showed encouraging results but very high study heterogeneity precluded strong recommendations for pathway use [20]. Recent work using an enhanced recovery after surgery (ERAS) protocol for patients undergoing resection with free flap reconstruction found reductions in LOS but no differences in complications among ERAS managed patients [21].

Long-term outcomes from formal quality management programs are lacking in the head and neck literature. All of the cited studies report results from short-term projects usually lasting from 1 to 3 years. Furthermore, none of the current studies describe the steps required to design and implement a quality management program. By reporting long-term results as well as details on program design and implementation we believe the uptake of care pathways will increase.

The current pathway reported in this study has some significant gaps. There are no data reported on pain control, management of postoperative nausea and vomiting, prehabilitation or details of mobilization after surgery. These important gaps are being closed with recent pathway modifications and we anticipate improvements in future outcomes. Stable support for the quality management program is an important enabler of continuous pathway improvement.

During the time period covered by this study the clinical volumes in our program increased. This was primarily due to changes in regional referral patterns and an increase in the number of patients requiring major surgery with free flap reconstruction. Although it is possible that this increase in surgical volume explains the improved outcomes we do not believe this is the case. The surgical, intensive care and nursing teams were unchanged throughout the study, the surgical techniques were consistent and the only practice change implemented was the care pathway approach articulated in this study. We therefore believe the care pathway is responsible for the reported changes in outcome.

This study also has some important limitations. We report results from a single institution and this might hamper generalizability of the results. However, the cohort size is large and the data were prospectively collected therefore making the results more generally applicable. There were also data missing on 30 patients. This is less than 10% of the overall cohort and we do not believe this impacted the results.

Strengths of the study include prospective data collection and broad eligibility criteria making the results more applicable to other programs. The parsimonious data collected is also a strength. Implementing and sustaining a quality management program requires significant time, resources and an engaged team of providers. Limiting the data elements to the “vital few” is a good strategy to improve feasibility and cost-effectiveness of the program. This approach also makes program implementation more accessible in smaller programs or in resource-challenged environments.

## Conclusions

In this study we describe the outcomes achieved from a sustained quality management program for patients undergoing major head and neck resection with free flap reconstruction. Outcomes, especially pneumonia rate, time to mobilization, time to decannulation and length of stay collected over a 7-year period show consistent improvement when compared to a baseline cohort. We believe that such an approach is an important strategy to maintaining excellent clinical performance in complex and resource-challenged healthcare environments.

## Data Availability

The data that support the findings of this study are available from the authors but restrictions apply to the availability of these data, which contain identifiable information, for the current study, and so are not publicly available. Data are however available from the authors upon reasonable request and with permission of the office of the privacy commissioner of Alberta.
